# Patterns and Determinants of Attitudes towards Genetic Risk of Cancer: Case Study in a Malaysian Public University

**DOI:** 10.1155/2018/4682431

**Published:** 2018-07-19

**Authors:** Edmund Ui-Hang Sim, Su-Hie Ting

**Affiliations:** ^1^Faculty of Resource Science and Technology, Universiti Malaysia Sarawak, 94300 Kota Samarahan, Sarawak, Malaysia; ^2^Faculty of Language and Communication, Universiti Malaysia Sarawak, 94300 Kota Samarahan, Sarawak, Malaysia

## Abstract

Genetic risk to cancer is a knowledge largely confined to experts and the more educated sectors of the developed western countries. The perception of genetic susceptibility to cancer among the masses is fragmented, particularly in developing countries. As cancer diseases affect developing countries as much as developed nations, it is imperative to study perception and reception of genetic risk to cancer in Southeast Asia. Here, we report on a novel case study to gauge the awareness and attitudes towards genetic determination of cancer among the undergraduates of a Malaysian public university. A total of 272 university undergraduate students completed an online questionnaire. On causes of cancer, the respondents believed that cancer is caused by lifestyle and environmental factors, but those with science background were more likely to associate it with genetic factors. The results on awareness of genetic profiling of cancer risk showed that there are significant differences between those with science and nonscience background but there are no significant differences for gender and socioeconomic background. As for attitudes towards cancer risk, female respondents, those from middle socioeconomic status and science background, are more likely to believe in genetic determinism of cancer. The findings have implications on target population segmentation in strategic health communication on cancer.

## 1. Introduction

The belief that cancer is essentially a genetic disease is no longer just a viewpoint but a fact to cancer biology experts since the beginning of the 21st century [1, 2]. Before the end of this century's first decade, cumulative findings on genetic or molecular profiling of cancer have prompted molecular biologists to support efforts in the quest for accurate diagnostic and treatment platforms [3–6]. This has led to an emergence of translational applications ranging from development and utility of big data in cancer [7, 8] to genetic testing avenues and associated policy in practice recommendations, particularly in the USA [9]. Thus far, it seems that awareness of genetic determinants in cancer is largely confined to cancer biologists, oncologists, and informed healthcare providers. The acceptance of this in the context of breast cancer risks is more pronounced among geneticists rather than oncologists [10]. It may, therefore, be necessary to study and gauge the perception and reception of genetic risks for cancer from the general public comprising every strata of the society.

Research on the public perception of genetic determinism and perceived control of health for the potential purpose of educational communication is not unprecedented. One such study revealed that the genetic susceptibility beliefs (threats) motivate behavioural response in health control, while beliefs in the molecular nature of disease (essentialism) produce an opposite response [11]. Insofar as findings related to perception of genetic predisposition to cancer, indirect and direct pieces of evidence have been made available. Indirect assessment of genetic factors as cancer risk can mostly be obtained from studies that look into people's attitudes and receptivity of genetic testing for cancer susceptibility. One such study done in the US [12] circumstantially revealed a high awareness and acceptance of genetics as an underlying factor in cancer predisposition. On the other hand, evidence from a study conducted in Ireland that directly surveyed public discernment of cancer risk [13] showed that a majority of the respondents have inaccurate understanding of cancer risks. Noncommon and common risk factors were overestimated and underestimated, respectively. Genetics is among those factors that were overestimated.

Most studies on public perception of genetic risks in cancer are restricted to Europe and USA. Conclusion inferred in developed countries of the western hemisphere may differ in Asia and Africa due to disparity in cultural background, literacy level, and scientific development. Even within Europe, research on this is regionally fragmented and lacks coverage of the whole European Union community. Hence, global perception of genetic factors and its competition or combination with environmental influence in cancer susceptibility remains ambiguous. In many developing and underdeveloped countries, it is not even fully certain whether canonical risk factors (lifestyle and environment) are a public concern, let alone genetic risk. In a recent review study on cancer-related genetic and genomic testing, a lack of existing research on awareness and communication of this matter in minority and underserved communities is revealed [14]. The case of Malaysia alone is an indication of general public ignorance. Samat and coworkers' [15] survey of 544 urban and educated Malaysians showed that 68% have heard of cancer and have some knowledge of cancer risk factors. Other studies have shown deficiency in knowledge of warning signs and risk factors for breast cancer [16–19], colorectal cancer [20], oral cancer [21–23], and cervical cancer [24–26]. In Malaysia, an awareness study on genetic risk and profiling of cancer has never been conducted before this.

Herein, we report a novel case study on the perception and reception of genetic risk in cancer among undergraduate students of a Malaysian public university. Our study examined patterns and determinants of attitudes towards genetic risk of cancer so that misconceptions can be addressed through health communication efforts.

## 2. Materials and Methods

The case study involved 272 undergraduate students in a Malaysian public university. As part of a larger research programme, this study represents the first phase where university students are selected in order to target the more educated sectors of the community.

The sociodemographic distribution of the respondents is shown in Table 1. A majority of the respondents, with an average age of 22, were female (76.5%) and from the science educational background in secondary school (80.1%). They were in their first-to-final year in their undergraduate degree programmes. The socioeconomic status of the respondents was almost equally divided between low class (53.7%) and middle class (46.3%). None of the respondents reported a monthly household income exceeding RM30,000 (high class).

The online questionnaire, prepared in English, consisted of three sections. The first section contained items on sociodemographic variables such as age, gender, ethnic group, secondary school background, socioeconomic status (average household monthly income), and location of residence. The second section included four 5-point Likert scale items on causes of cancer (genetic, lifestyle, and luck, Table 2). The responses were scored 1 to 5 (1 for strongly disagree, 2 for disagree, 3 for neutral, 4 for agree, and 5 for strongly agree). The third section, also employing the same 5-point Likert scale, examined knowledge and attitudes towards genetic profiling of cancer risk. This section comprised 13 items on awareness of genetic profiling (4 items), attitudes towards cancer risks (6 items), and communication of genetic risks of cancer (3 items). Items are shown in Table 3.

Students were informed of the study and assured of the privacy and confidentiality of their responses in reports arising from the data they provided. Students who did not wish to participate in the study did not fill in the questionnaire. Students who consented to participate in the study went to the survey questionnaire, entitled “Public Perception of Biological (Genetic) Susceptibility to Cancer,” built using the free online Google Form interface/platform (URL: https://docs.google.com/forms/d/1Zk0NbVTqYdAXsUmUyU9HsZxvZDZo6acTligUqaztdj8/edit). They filled in the questionnaire during their free time until the end of the semester.

In data analysis, the sociodemographic variables were analysed as independent variables and the dependent variables were beliefs on causes of cancer and knowledge and attitudes towards genetic profiling of cancer risk. The results were reported as means and standard deviation. Comparisons of differences for gender, secondary school background (science* versus *art stream), and socioeconomic status were performed using Student's* t*-test.

## 3. Results

### 3.1. Beliefs on Causes of Cancer

Three causes of cancer were explored: genetic, lifestyle, and random causes (luck). The lifestyle cause was examined using one positive item (diet control and physical exercise) and another negative item (smoking, alcohol consumption, and radiation exposure). The results showed that the respondents agreed with genetics and lifestyle as predisposition factor but disagreed that it was due to luck (Table 2).

There were no significant differences between female and male respondents in their beliefs on the causes of cancer. There were also no significant differences for respondents with low and middle socioeconomic status. However, respondents from science and arts stream were significantly different in their beliefs on whether cancer diseases can be influenced by genetic factors (p < 0.01). The science respondents (M=4.3) were more aware of genetic predisposition to cancer risk than the arts respondents (M=3.7). Science stream respondents had greater knowledge of science and presumably are more attuned to the biological nature of diseases.

### 3.2. Awareness of Genetic Profiling for Cancer Risk

The respondents were aware of genetic profiling for cancer risk (Table 3). They agreed that cancer diseases can be genetically profiled (M=3.6) and that a person's genetic profile can be used to check whether they are at risk of cancer diseases (M=4.1). The respondents knew that there is a lack of research on genetic profiles of various cancer diseases (M=3.5) and that genetic biomarkers have been developed for use in cancer risk prediction (M=3.6). There were no significant differences between female and male respondents in their awareness of genetic profiling of cancer risk. There were also no significant differences between respondents from low and middle socioeconomic background. Regarding the relationship between secondary school educational background and awareness of genetic profiling of cancer risk,* t*-tests showed significant differences between science and arts stream respondents for three out of four aspects examined. The mean scores showed that the science stream respondents expressed stronger agreement. The only item for which there were no significant differences was on lack of research on genetic profiles of various cancer diseases (p > 0.05).

### 3.3. Attitudes towards Cancer Risk

Most respondents disagreed that research on genetic risks of cancer is a waste of public funding (M=1.5) and the researchers' time and effort (M=1.4) (Table 4).* t*-test results showed that significant differences for science and arts stream respondents on both aspects (p < 0.01) and a significant difference between female and male respondents on whether research on genetic risks of cancer are a waste of the researcher's time and effort. Female respondents (p=0.02) and those with middle socioeconomic status (p < 0.01) are more likely to believe that it is important for researchers to study genetic risks of cancer. Those with middle socioeconomic status are also more likely to disagree that research on genetic risks of cancer is a waste of public funding (p < 0.01).

On genetic risks in cancer, most of the respondents agreed that it is important for them to know about their risk (M=4.5) and disagreed that it is only important for people who suffer or are affected by cancers (M=2.0).* t*-tests results showed that attitudes towards cancer risk differed significantly with three sociodemographic variables (Table 4). Science stream respondents and those with middle socioeconomic status attributed more importance for everyone (including themselves) to know their risk of getting cancer. Compared to male respondents, female respondents felt more strongly that people who do not suffer from cancer also need the cancer risk information.

On the financial burden of cancer, most of the respondents agreed that it is important for everyone to know about the financial implications of cancer (M=4.3) and disagreed that the information is only important to cancer patients (M=2.0).* t*-tests showed that there were significant differences in the importance of the financial risk of cancer to science and arts stream respondents (p < 0.05). The science stream respondents felt more strongly about the need for everyone and not just cancer patients to know the treatment expenses and loss of income potentially accrued. The female respondents expressed stronger disagreement that the financial risk information is only important to cancer patients.

The respondents were unsure whether genetic risks of cancer had been communicated to the public in Malaysia (M=3.1, Table 5). The female respondents could not form an opinion on whether genetic risks of cancer had been communicated to the public, whereas the male respondents were marginally positive that this had taken place. However, they felt that the risks should be communicated to the public (M=4.4) and, at present, the information is too technical for the public to understand (M=3.7). There were significant differences on their perceptions on these two aspects of cancer risk communication due to the respondents' educational background (p < 0.05). The science stream and middle class respondents expressed stronger beliefs on the need to communicate genetic risks of cancer to the public. Further, the science stream respondents felt more strongly that the genetic cancer risk information can only be understood by medical specialists and cancer geneticists.

## 4. Discussion

Our findings on perception of cancer causes do not deviate from those done in the US [12] and Europe–Ireland [13]. Generally, awareness of genetic factor as one of the main determinants is evidently high. Gender and socioeconomic status were not a factor to this, and luck is rejected. Our data, however, is a mismatch with numerous studies done in Malaysia [15–26] that reported the awareness level of cancer risk factors as moderate to low. Comparing our efforts with work by others locally may be unsuitable as the issue of genetic determinism is not prompted or introduced in their surveys. Furthermore, we surveyed the more educated sector of society, that is, university undergraduate students.

We reveal that respondents with science-based educational background are more aware compared to those from art-based training. Studies done in the US [12] and Europe [13] did not have this information because this aspect was not incorporated in their research. The understanding that cancer disease can be genetically profiled is generally high among the respondents in our study. This extends to the awareness of the existence of cancer biomarkers. It can be attributed to the fact that a majority of our respondents have educational background in the science. Essentially, this attitude is similar to results surveyed in the US [12] that reported high public acceptance of genetic tests for cancer risk. In fact, there is a high agreement among the respondents in our study on the importance for everyone to test and know their genetic risks to cancer. In our case, we also reveal that those from science-based training tend to agree more with this compared to respondents with art-based educational background. This attitude is also gender-biased because women tend to be more positive towards tests on genetic risks than men. In a study on women with breast cancer, positive reception to a genetic risk profile is due to the perceived benefits in obtaining clarification and justification for the diagnosis and risk management decisions implemented on them and also an explanation of their own risks and that of other family members [27]. Whether this is the same or different for men with prostate cancer in particular and other cancers in general remains to be explored.

Women and those from the science stream also felt strongly that research on genetic risks of cancer is not a waste of time, effort, and money. Such strong acceptance by women is notably common, as famously exemplified by the actions (bilateral mastectomy) of an American actress, Angelina Jolie, in reducing her risks of breast cancer after discovering she had* BRCA1* gene mutation. The “Angelina effects” that followed have not only created a global surge in public awareness of breast cancer susceptibility genes and familial cancer but have significantly increased similar behavioural change among women [28–30]. In the Asian context, there is a sharp rise among Korean women in the uptake of genetic testing and the resolve towards risk-reducing measures (salpingo-oophorectomy and contralateral prophylactic mastectomy) among those found to have genetic mutations associated with hereditary breast and ovarian cancer [31]. Such effects have yet to be explored in Malaysia. Our findings also reveal a general agreement among respondents that public awareness of financial burden to the cancer problem is pertinent. Notably, this viewpoint is stronger in females and those from science background in our study.

Respondents in our survey generally believed that the public communication of genetic susceptibility to cancer is necessary. This is more apparent among those from the science stream, despite their opinion that the matter may be too technical for public consumption. Hence, research efforts to simplify such information for effective communication to the public are implied. Recent literature research on cancer-related genetic/genomic testing has exposed the inadequacy of effective provider-patient communication due, in part, to a lack of systematic studies in health literacy and communication [14]. This lack of research in communication practices by health professionals on cancer genetic risk and testing is further substantiated in a study by Hamilton et al. [32].

In terms of behaviour theory on genetic determinism of diseases, our findings did not converge with explanation by Parrott et al. [11]. According to Parrott et al. [11], behavioural response to threats (genetic susceptibility) is positive while to essentialism (molecular nature of disease) is negative. Limited to data on cancer (and not health in general), we find that those who believed in the genetic causes of disease accepted genetic susceptibility information and supported the relevance of matters related to genetic testing. Information on whether they will actually go for such tests remains to be further pursued. In our case, within the context of undergraduate students in the university studied, perhaps knowledge of biological fate to cancer diseases is not separated into genetic risk and the academic contemplation of cancer genetics. In other words, the threat belief is influenced by essentialism model, which then motivates behavioural change. Clearly, studies in more Malaysian universities are warranted to specifically test this theory in the local context before we can suggest a refinement to the hypothesis by Parrott et al.

## 5. Conclusion

Based on our case study in a Malaysian public university, perception of genetic risk to cancer is generally high among the group of undergraduate students surveyed. Female respondents and those from middle socioeconomic and science background were stronger in their belief on this. Albeit a confined study sample, our findings have preliminary implications for sustainability of genetic profiling efforts (research and application); implementation of genetic testing; accessibility of financial burden information; and effective health communication strategy for the cancer problem in the Malaysian context. Similar study of a larger scale should be conducted to enhance understanding of cancer risk factors for more effective health communication efforts.

## Figures and Tables

**Table 1:** Sociodemographic distribution of the sample of university students (N=272).

Variable		*n *(%)
Age	Mean in years (SD)	22.2 (1.2)
	Range	20-25
Gender (n %)	Female	208 (76.5%)
	Male	64 (23.5%)
Ethnic group (n %)	Malay	144 (52.9%)
	Chinese	45 (16.6%)
	Indigenous	75 (27.6%)
	Indian	8 (2.9%)
Secondary school background (n %)	Science	218 (80.1%)
	Art	54 (19.9%)
Socio-economic status (n %)	Low class (below RM3,000 per month)	146 (53.7%)
	Middle class (RM3,000-RM30,000)	126 (46.3%)

**Table 2:** Mean and standard deviation for beliefs on causes of cancer (N=272).

		Mean for science (n=218)	Mean for arts (n=54)	Overall Mean	Overall SD
(1)	Cancer diseases can be influenced by genetic factors.	4.3^a^	3.7^a^	4.2^a^	0.838
(2)	Lifestyle changes (e.g., diet control and physical exercise) can prevent cancers.	3.9	4.0	4.0	0.864
(3)	People get cancer because of external (environmental) factors such as smoking, alcohol consumption, and exposure to radiation and chemical agents.	4.3	4.1	4.2	0.760
(4)	People get cancers because they are unlucky.	2.0	1.8	2.0	1.057

*Note.* 1 for strongly disagree, 2 for disagree, 3 for neutral, 4 for agree, and 5 for strongly agree.

^a^Differences between science and arts stream respondents were significant at p < 0.01.

**Table 3:** Mean and standard deviation for awareness of genetic profiling of cancer risk (n=272).

		Mean for science (n=218)	Mean for arts (n=54)	Overall Mean	Overall SD
(1)	Cancer diseases can be biologically profiled, i.e., genetic fingerprints can be constructed to discriminate/distinguish between those who are at risk and those who are not.	3.8^a^	2.9^a^	3.6^a^	0.983
(2)	Do you agree that a person's genetic profile can be used to check whether he/she is at risk of cancer diseases?	4.2^a^	3.7^a^	4.1^a^	0.791
(3)	There is a lack of research on biological (genetic) profiles of various cancer diseases.	3.5	3.4	3.5	0.863
(4)	Are you aware that biological profiles based on genetic biomarkers have been developed for use in cancer risk prediction?	3.7^a^	3.2^a^	3.6^a^	0.978

*Note.* 1 for strongly disagree, 2 for disagree, 3 for neutral, 4 for agree, and 5 for strongly agree.

^a^Differences between science and arts stream respondents were significant at p < 0.01.

**Table 4:** Mean and standard deviation for attitudes towards cancer risk (n=272).

		Stream		Gender		Socio-economic status		Overall Mean	Overall SD
		Science	Arts	Female	Male	Low	Middle		
		(n=218)	(n=54)	(n=208)	(n=64)	(n=146)	(n=126)		
(1)	Research on biological risks of cancer is a waste of public funding.	1.4^a^	2.0^a^	1.5	1.7	1.7^c^	1.3^c^	1.5^a,c^	0.871
(2)	Research on biological risks of cancer is a waste of time and effort of researchers.	1.3^a^	1.9^a^	1.4^b^	1.7^b^	1.6^c^	1.2^c^	1.4^a,b,c^	0.780
(3)	It is important for me to know about my genetic risks in cancer.	4.6^a^	4.3^a^	4.6	4.4	4.4^c^	4.6^c^	4.5^a,c^	0.625
(4)	Do you agree that knowing genetic risks of cancers is only important for people who suffer or are affected by cancers?	1.9^a^	2.4^a^	1.9^b^	2.3^b^	2.1^c^	1.8^c^	2.0^a,b,c^	1.193
(5)	It is important for everyone to know about the financial burden of cancers (e.g., treatment expenses and loss of income).	4.3^a^	4.0^a^	4.3	4.2	4.2	4.4	4.3^a^	0.820
(6)	Information on financial burden of cancers is only important to those who suffer or are affected by cancers.	1.9^a^	2.4^a^	1.9^b^	2.5^b^	2.0	2.1	2.0^a,b^	1.217

*Notes.* 1 for strongly disagree, 2 for disagree, 3 for neutral, 4 for agree, and 5 for strongly agree.

^a^Differences between science and arts stream respondents are significant at p < 0.01.

^b^Differences between female and male respondents are significant at p < 0.05.

^c^Differences between respondents with low and middle socioeconomic status are significant at p < 0.05.

**Table 5:** Mean and standard deviation for communication of genetic risks of cancer (n=272).

		Stream		Gender		Socio-economic status		Overall Mean	Overall SD
		Science	Arts	Female	Male	Low	Middle		
		(n=218)	(n=54)	(n=208)	(n=64)	(n=146)	(n=126)		
(1)	Biological (genetic) risks of cancers have not been communicated to the public in my country.	3.1	3.0	3.0^b^	3.4^b^	3.1	3.1	3.1^b^	1.055
(2)	Biological (genetic) risks of cancers should be communicated to the public in my country.	4.5^a^	3.9^a^	4.4	4.3	4.2^c^	4.5^c^	4.4^a,c^	0.798
(3)	At the moment, information on biological (genetic) risks of cancers is too technical and can only be understood by medical specialists and cancer geneticists.	3.8^a^	3.4^a^	3.7	3.8	3.7	3.8	3.7^a^	0.986

*Notes.* 1 for strongly disagree, 2 for disagree, 3 for neutral, 4 for agree, and 5 for strongly agree.

^a^Differences between science and arts stream respondents are significant at p < 0.01.

^b^Differences between female and male respondents are significant at p < 0.05.

^c^Differences between respondents with low and middle socioeconomic status are significant at p < 0.05.

## Data Availability

The data used to support the findings of this study are available from the corresponding author upon request.
